# Dilated Thoracic Esophagus Presenting with Painful Progressive Persistent Dysphagia and Leukocytosis of Unknown Origin

**DOI:** 10.7759/cureus.1851

**Published:** 2017-11-16

**Authors:** Kyawzaw Lin, Jamil Shah, Emmanuel Ofori, Vahe Shahnazarian, Madhavi Reddy

**Affiliations:** 1 Medicine, The Brooklyn Hospital Center; 2 Internal Medicine, The Brooklyn Hospital Center; 3 GI Department, The Brooklyn Hospital Center; 4 Gastroenterology & Hepatology Fellow, The Brooklyn Hospital Center

**Keywords:** esophageal cancer, dysphagia, dilated esophagus, leucocytosis of unknown origin, gastroenterology, dilated thoracic esophagus

## Abstract

Esophageal cancer is the eighth-most common cause of cancer-related mortality worldwide. The most common presenting symptom in advanced distal esophageal cancer is the sensation of sticking food, but it may sometimes present with bleeding and related complications, or asymptomatic leukocytosis. We present the case of a 77-year-old afebrile man with chronic alcoholism and a dilated thoracic esophagus with painful, progressive, and persistent dysphagia and leukocytosis of unknown origin.

A 77-year-old man with a past medical history of hypertension and colonic cancer status post right hemicolectomy (surveillance negative) presented to the emergency department with painful, progressive, persistent, and worsening dysphagia for the past three weeks. It was associated with an unintentional weight loss of ten pounds in one month and nausea with non-bilious and non-bloody vomiting for several days. He denied fever, diarrhea, hoarseness of voice, change in bowel movement, hematemesis, hematochezia, melena, orthopnea, dyspnea at rest, palpitation, and abdominal pain. A chest x-ray (lateral view) showed debris in a dilated thoracic esophagus with fluid. An esophagogram showed a 10 x 3 cm obstructive mass with irregular mucosa within the proximal esophagus from the thoracic vertebra levels four to ten. A computed tomography scan of the chest with contrast showed long segment dilatation of the upper and mid-thoracic esophagus with generalized circumferential thickening of the distal esophagus. He was empirically on cefazolin and metronidazole but later switched to piperacillin, tazobactam, and fluconazole. Cardiac risk stratification was done for an esophagogastroduodenoscopy. However, the patient and the family opted for palliative care and agreed to a do-not-resuscitate/do-not-intubate status.

In esophageal cancers, tumor-related leukocytosis and neutrophilia are common presentations. However, there is no standardized routine screening test for esophageal cancers. Thus, when asymptomatic afebrile elderly patients present with leukocytosis of unknown origin, clinicians should have suspicions of occult malignancy such as esophageal cancers, gastric cancer, and pancreatic cancer.

## Introduction

Esophageal cancer is the eighth most common cause of cancer-related mortality worldwide with 456,000 new cases and 400,200 annual deaths in 2012 [1]; the five-year survival rate is only 15% to 20%. Even after surgical intervention, the morbidity rate is 87%, and the mortality rate is dismally high at 26% [2]. Currently, gastric cardia adenocarcinoma and adenocarcinoma of the distal esophagus are on the rise, accounting for 50% of new cases. Globally, esophageal squamous cell carcinoma (ESCC) is reported to account for 78% of esophageal cancer cases and is most prevalent in South-Eastern and Central Asia. The incidence of esophageal adenocarcinoma (EAC) surpasses ESCC in the UK, US, Canada, and Norway [3]. The most common presenting symptom for advanced distal esophagus is “the reported sensation of sticking of food in the esophagus”, but it may sometimes present with bleeding or related complications.

## Case presentation

**Figure 1:**
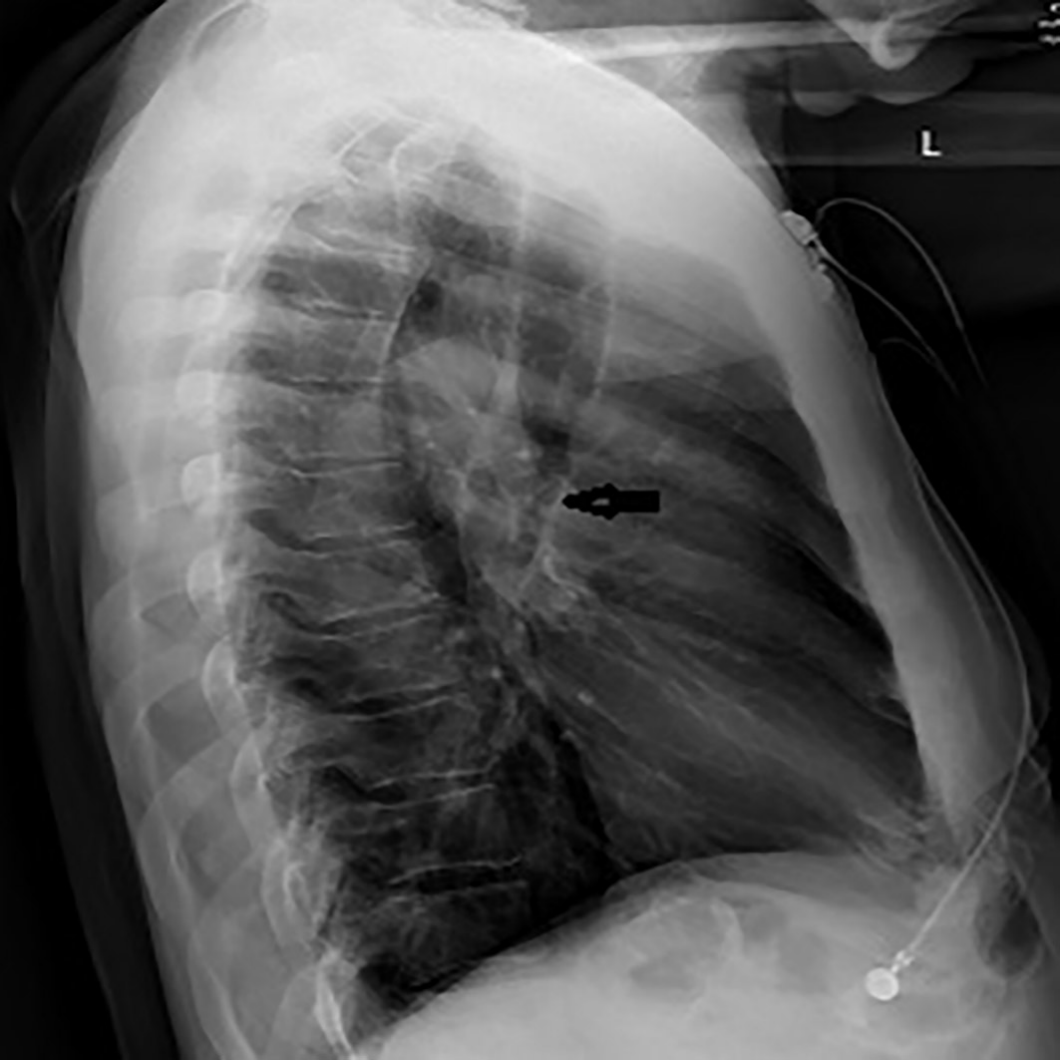
Chest X-ray (lateral) view showed debris in a dilated thoracic esophagus with fluid level (black arrow).

**Figure 2:**
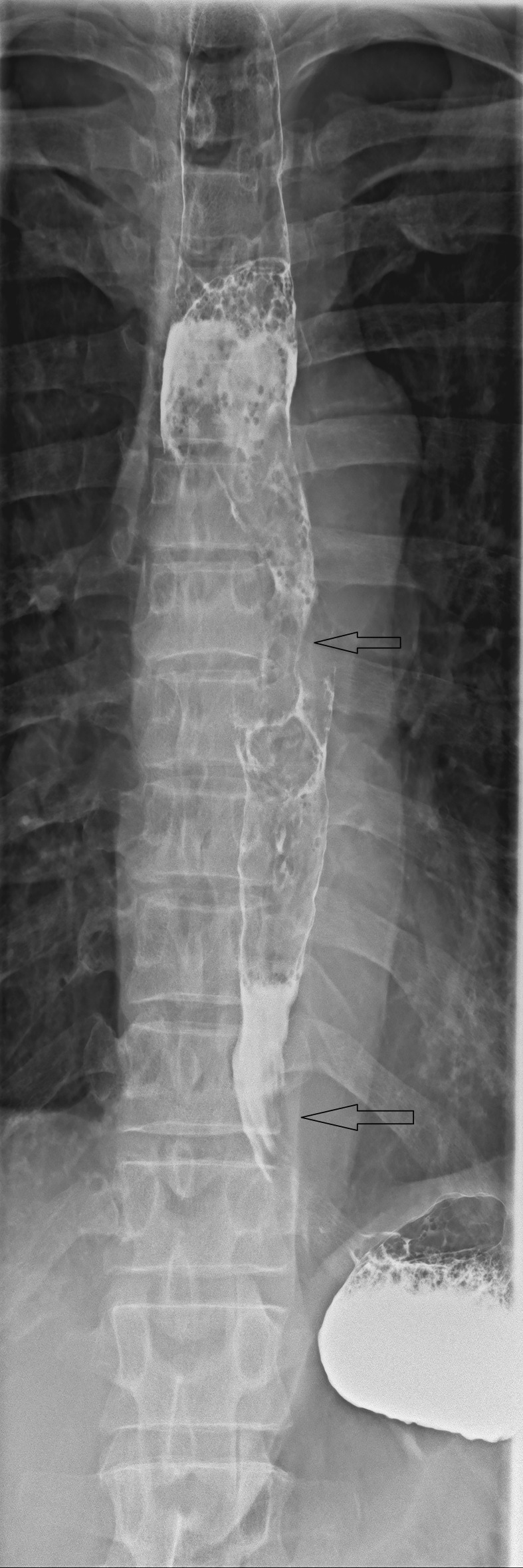
Esophagogram showed 10 x 3 cm obstructive mass with irregular mucosa (arrows) within the proximal esophagus from T4 -T10 level. T4: 4^th^ thoracic vertebra T10: 10^th^ thoracic vertebra

**Figure 3:**
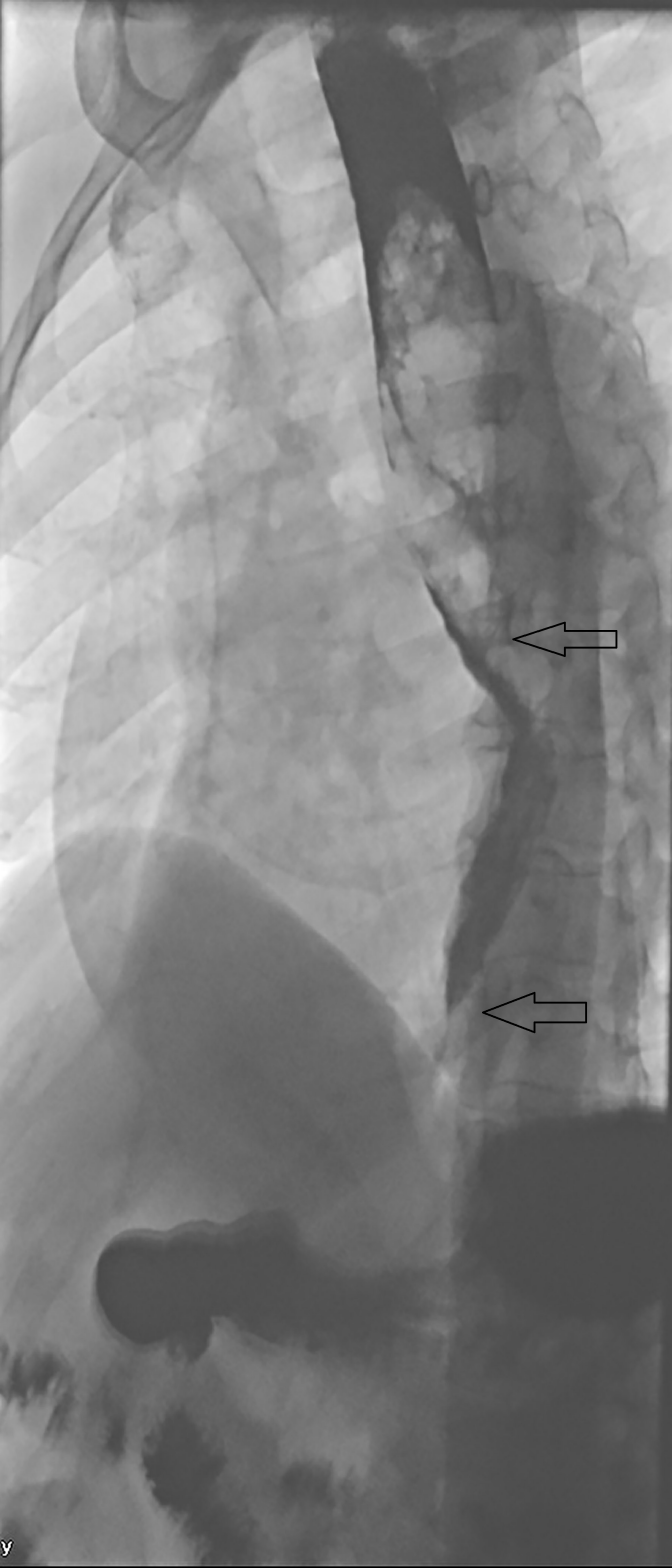
Esophagogram showed 10 x 3 cm obstructive mass with irregular mucosa ( arrows) within the proximal esophagus from T4 -T10.

**Figure 4:**
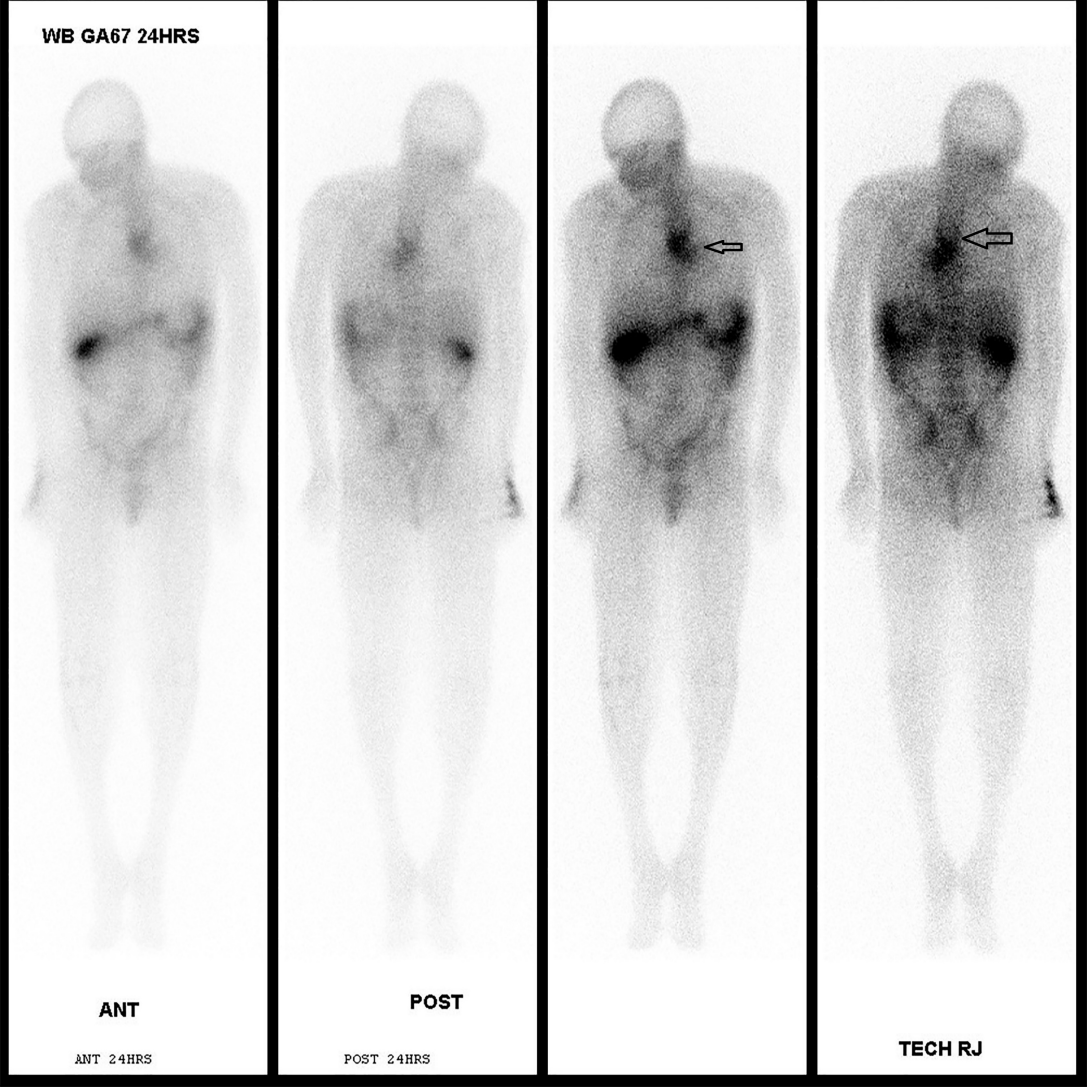
Gallium scan for whole body showed moderate to marked abnormal radioactivity at the mid esophagus, extending inferolaterally to the left with esophageal malignancy with focal extra esophageal invasion or esophageal abscesses with or without underlying malignancy.

**Figure 5:**
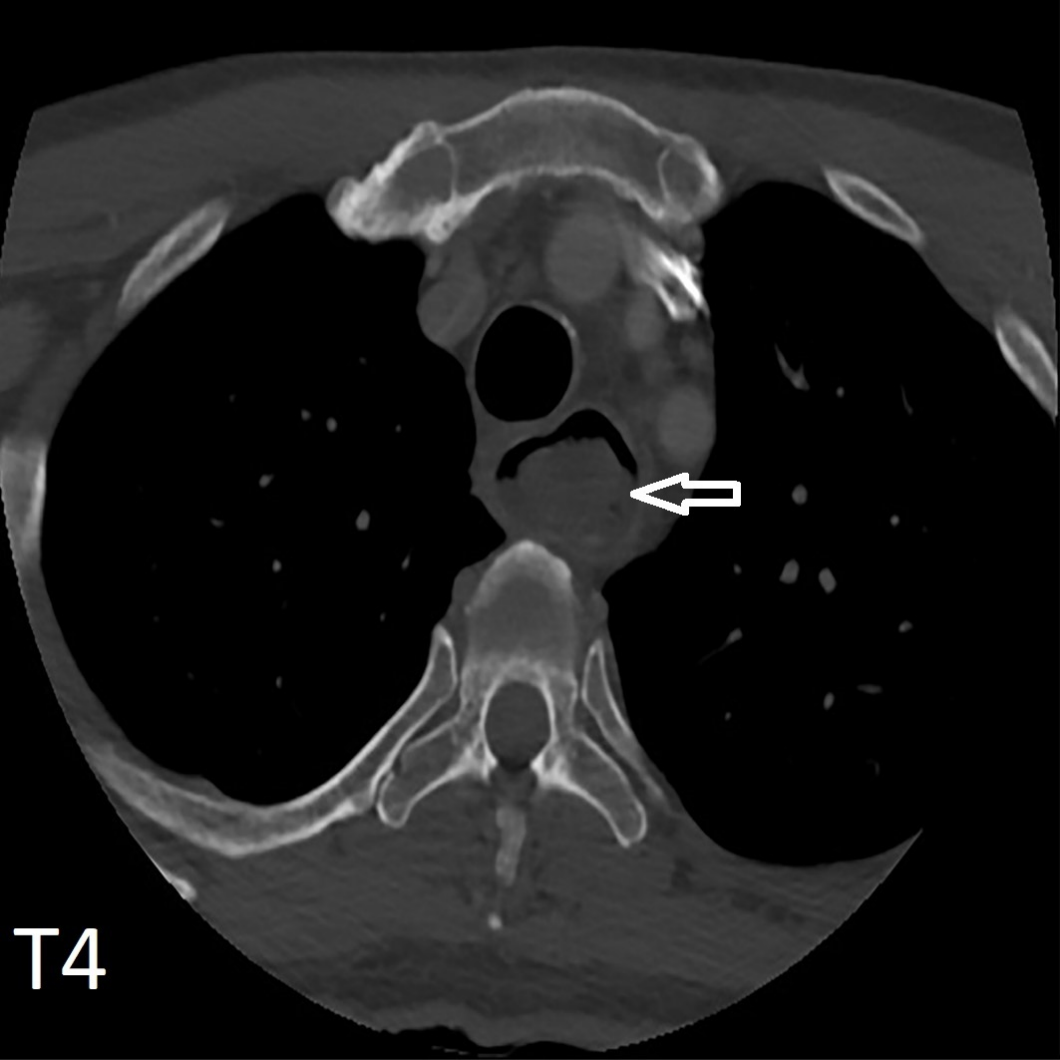
Computed tomography scan (chest) with contrast at T4 level showed long segment dilation of the upper and mid thoracic esophagus with intraluminal heterogeneous material with generalized circumferential thickening of the distal esophagus. T4 (4^th ^thoracic vertebra)

A 77-year-old man with a past medical history of hypertension and colonic cancer status post right hemicolectomy (surveillance negative) presented to the emergency department with painful, progressive, persistent, and worsening dysphagia for the past three weeks. He had solid to liquid dysphagia over three months as well. He felt food getting stuck in his neck and felt nauseous when he tried to drink and would spit up some liquid. He was prescribed a few courses of antibiotics for a sore throat, but the condition did not improve. The sore throat was associated with an unintentional weight loss of ten pounds in one month and nausea with non-bilious and non-bloody vomiting for several days. He denied fever, diarrhea, hoarseness of voice, change in bowel movement, hematemesis, hematochezia, melena, orthopnea, dyspnea at rest, palpitation, and abdominal pain. A chest x-ray (lateral view) showed debris in a dilated thoracic esophagus with fluid (Figure 1). An esophagogram showed a 10 x 3 cm obstructive mass with irregular mucosa within the proximal esophagus from the thoracic vertebra levels four to ten (Figure 2, Figure 3). A whole-body gallium scan showed moderate to marked abnormal radioactivity at the mid-esophagus with an impression of esophageal malignancy with focal extra-esophageal invasion or esophageal abscesses with or without underlying malignancy (Figure 4). A computed tomography (CT) scan of the chest with contrast showed long-segment dilatation of the upper and mid-thoracic esophagus with intraluminal heterogeneous material with generalized circumferential thickening of the distal esophagus (Figure 5). A CT scan of the abdomen and pelvis showed no gross pathology. He was empirically on cefazolin and metronidazole but later switched to piperacillin, tazobactam, and fluconazole. His stress test was positive for ischemia/infarct. A transthoracic echocardiogram revealed a left ventricular ejection fraction of 55%, trace regurgitation, small pericardial effusion, and mildly dilated proximal ascending aorta. The results of his regadenoson injection were within reference ranges, and his cardiac catheterization results were unremarkable. However, the patient and the family opted for palliative care and agreed to a do-not-resuscitate/do-not-intubate status.

## Discussion

Over the past three decades, the incidence of esophageal cancer has risen due to an increase in the incidence of EAC cases. The mean annual increase in incidence is nearly 5% in Western Europe while it is 7% to 8% in the US. The well-known risk factor for EAC is gastroesophageal reflux disease (GERD) along with Barrette's esophagus [4-5]. Another predisposing factor is obesity, accounting for one-third of cases worldwide and nearly 43% of cases in developed countries. Recently, a high incidence in EAC is reportedly associated with Helicobacter pylori eradication as the bacteria lowers gastric acidity and acid reflux; its eradication is followed by weight gain [6]. However, for ESCC, both alcohol abuse and smoking are risk factors due to the associated chronic irritation and esophageal mucosa inflammation.

Cancer cachexia from weight loss due to a loss of skeletal and fat tissue is associated with a high mortality and accounts for 20% of cancer-related deaths [7]. The incidence of weight loss in esophageal cancer is nearly as high as the weight loss associated with pancreatic and stomach cancers. Significant pre- and post-treatment weight loss are also associated with high mortality.

In esophageal cancers, granulocyte-colony stimulating factors are activated via the paracrine effect and the intratumoral chemotaxis effect, which leads to tumor-related leukocytosis and neutrophilia [8].

The neutrophil-to-lymphocyte ratio (NLR) is correlated with tumor regression as well as poor survival. An NLR >3.5 is associated with worse overall survival (OS), progressive free survival (PFS), and local regional control. Significant factors related to worse OS include high white blood cell counts (especially neutrophil and monocytes), thrombocytosis, high-performance score (PS), and tumor length >7 cm on diagnosis. However, leukocytosis and neutrophilia are independent factors with poorer OS and worse PFS. The length of tumor on endoscopy has an enormous impact on OS and distant metastatic control (DMC). The tumor-nodes-metastases classification is not very accurate in predicting the outcomes of esophageal cancers [9]. Systemic inflammatory markers such as C-reactive protein (CRP), lymphocyte counts, neutrophil counts, and platelet counts can be considered predictive factors for esophageal cancers [10].

For inoperable esophageal cancers, the Glasgow Prognostic Score (GPS), NLR, CRP, and albumin in combination with conventional staging increased the survival rate. With locally advanced esophageal cancers with chemoradiation therapy, OS and PFS are associated with variations in the platelet-to-leucocyte ratio (PLR) and the NLR [9]. In ESCC with curative treatment, preoperative leukocytosis might relate to a worse OS and DMC [10].

Currently, there are no standardized routine screening tests available for esophageal cancers. Screening should focus on high-risk groups with chronic GERD and comorbid conditions such as advanced age, male sex, obesity, and family history; ESCC has family segregation in certain regions such as China. Esophageal cancer is still one of the higher mortality cancers. Thus, clinicians should consider treatment modalities with endoscopic mucosa resection in the early stage and surgical resection with chemo-radiation therapy in patients with locally advanced tumors (as seen in our patient). Clinicians should consider palliative care for patients with metastatic or unresectable tumors.

## Conclusions

In esophageal cancers, tumor-related leukocytosis and neutrophilia are common presentations due to the paracrine effect and the intra-tumor chemotaxis effect. Leukocytosis and neutrophilia are independent factors with poorer OS and worse PFS. Unfortunately, no standardized routine screening test for esophageal cancers has been established with proven benefits. Thus, as in our case, whenever asymptomatic afebrile elderly patients present with leukocytosis of unknown origin, clinicians should suspect occult malignancy such as esophageal cancers, gastric cancer, and pancreatic cancer.
